# Application of Anti-Müllerian Hormone in Regulation of Ovarian Function and Its Practical Relevance for Fertility and Embryo Production in Cows and Mares

**DOI:** 10.3390/vetsci13060552

**Published:** 2026-06-03

**Authors:** Andreas Vernunft, Dragos Scarlet

**Affiliations:** 1 Research Institute for Farm Animal Biology (FBN), Wilhelm-Stahl-Allee 2, 18196 Dummerstorf, Germany; 2 Institute of Veterinary Anatomy, Vetsuisse Faculty, University of Zurich, Winterthurerstrasse 260, 8057 Zurich, Switzerland; dragos.scarlet@uzh.ch

**Keywords:** AMH, cattle, horses, follicle development regulation, fertility, embryo production

## Abstract

In the female organism, Anti-Müllerian hormone (AMH) is produced exclusively by granulosa cells, particularly in secondary and early tertiary follicles. The amount of AMH released into the blood is proportional to the total number of follicles present in the ovaries and is therefore used to estimate the ovarian functional reserve. In both cattle and mares, a strong and highly repeatable association has also been demonstrated between AMH concentrations and antral follicle count. Therefore, AMH may serve as a biomarker with broad diagnostic utility in reproductive medicine in these species, as it reflects the functional ovarian reserve through its association with viable antral and preantral follicles. While AMH is already being used for clinical diagnostics in human reproductive medicine and has been extensively investigated, it has received little attention in applied veterinary practice so far. This review explores the applications of AMH for reproductive medicine in cattle and horses, monoovulatory farm animals in which assisted reproductive technologies are widely used. The objectives of this review are to (I) provide clinically relevant background information and a comparative discussion of (II) the role of AMH in follicular development, (III) its value as a fertility indicator, and (IV) its significance for embryo production in vivo and in vitro in cows and mares, with particular emphasis on practical relevance and potential applications.

## Simple Summary

Anti-Müllerian hormone (AMH) is produced by granulosa cells in the ovary and reflects the number of developing follicles, making it a reliable indicator of ovarian reserve. In cattle and mares, AMH concentrations show a strong and consistent correlation with antral follicle count, linking it directly to the pool of viable oocytes. This makes AMH a robust endocrine biomarker for assessing ovarian function. While AMH is well established in human reproductive medicine, its application in veterinary species has been limited. Current evidence suggests it has significant potential for evaluating reproductive capacity, predicting response to assisted reproductive technologies, and supporting embryo production programs. This review highlights its physiological role in follicular development and examines its diagnostic value for fertility and embryo yield in cows and mares, with emphasis on its practical integration into breeding and reproductive management strategies.

## 1. Introduction

Anti-Müllerian hormone (AMH) was described as early as 1947 as a testicular factor that controls the development of the male sex during embryonic development by suppressing the Müllerian ducts [1]. AMH is a dimeric glycoprotein that belongs to the transforming growth factor beta (TGF-β) family, whose members are actively involved in tissue growth and differentiation—functioning as either inhibitors or stimulators depending on cell type and developmental stage [2,3]. In the female organism, AMH is produced exclusively by granulosa cells, mainly in secondary and small antral tertiary follicles, but it can also be detected at lower levels in primordial follicles and large tertiary follicles [4,5]. It was first demonstrated in mice that AMH limits the activation of primordial follicles and helps to prevent their premature depletion. Furthermore, it also decreases the sensitivity of developing follicles to follicle-stimulating hormone (FSH) [6]. Therefore, the resting follicle pool and the follicles recruited for follicular waves are regulated by a self-limiting negative feedback mechanism. The amount of AMH released is proportional to the number of follicles present in the ovaries [7,8,9] and is therefore used to estimate the ovarian functional reserve.

The ovarian functional reserve is defined as the pool of resting, healthy, and recruitable primordial follicles in the ovarian tissue. It is fully established during fetal life and is therefore determined at birth. From then on, the ovarian reserve declines irreversibly, slowly but steadily over the course of life as a result of atresia and recruitment prior to ovulation [10]. Peripheral AMH levels in adult women, cows, and mares are considered to be largely stable and independent of the ovarian cycle [11,12,13,14]. However, when interpreting AMH concentrations in cattle, it must be noted that calves have very high AMH levels in the first months of life due to an early and transient activation of the hypothalamic–pituitary–adrenal (HPA) axis (also described as “mini-puberty”), and cows can show a sharp drop during the postpartum period, when ovarian silence can occur [15,16,17]. With the onset of menopause in women, the ovarian functional reserve is considered exhausted and AMH levels drop sharply [18]. In farm animals, the age-dependent decline is only of minor importance, as most of them reach the end of their productive lifespan long before their ovarian reserves are depleted. However, in old mares, a decline in ovarian activity and a drop in AMH levels can certainly be observed [19,20]. Interestingly, seasonal effects on ovarian activity (ovulatory summer season versus anovulatory winter season) can be mostly disregarded when considering AMH levels in mares [20].

**Figure 1:**
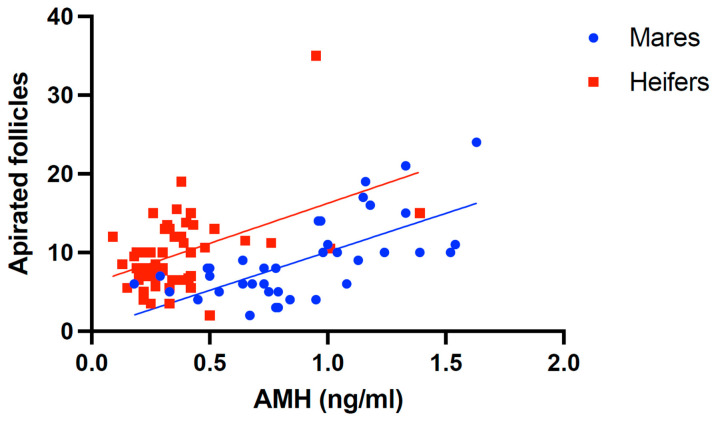
Relationship between plasma AMH concentrations and the numbers of aspirated follicles during ovum pick-up programs in heifers and mares showing comparable correlations. (According to Vernunft et al., 2011 [14] and Vernunft et al., 2015 [32]).

There is a high positive correlation between the ovarian functional reserve and the number of tertiary follicles that participate in the follicular waves in the ovarian cycle. Therefore, the antral follicle count (AFC) is also a reliable parameter for estimating the ovarian functional reserve, which can be determined by a careful ultrasonographic examination under field conditions without any laboratory testing [8,21]. Thus, when considering the relationship between ovarian functional reserve and fertility parameters, AMH and AFC are discussed in parallel. In cows and mares, a strong and repeatable correlation exists between AMH plasma concentrations and AFC (Figure 1). Strong linear regressions between plasma AMH levels and AFC have been described in various cattle breeds [8,22,23]. In mares, we demonstrated as early as 2011 that plasma AMH concentrations are positively associated with ovarian activity and antral follicle formation, exhibit intra-individual stability over time, and therefore represent a reliable biomarker for predicting follicular development capacity in ovum pick-up programmes [14]. In the meantime, the results have been confirmed by numerous other studies in other species [23,24,25,26,27,28,29,30,31].

Innate ovarian reserve is highly variable, offering opportunities to select for different ovarian reserves. In cattle, for example, 1920 to 40,960 oocytes were counted at the age of one year [8,33]. The ovarian functional reserve exhibits moderate to high heritability in this species, supporting its potential as a target for selective breeding [34,35,36]. Genomic heritability estimates are 0.36 for AMH concentrations [35], while AFC shows estimates of 0.25 in heifers and 0.31 in dairy cows [36]. Nevertheless, accumulating evidence indicates that extrinsic factors acting during prenatal life can significantly influence the ovarian reserve and subsequent AMH concentrations. For example, exposure to a high temperature–humidity index (THI) during the first trimester of gestation has been negatively associated with the size of the offspring’s ovarian reserve, as reflected by lower serum AMH concentrations and reduced AFC in animals conceived during summer compared with winter months [37]. However, this has not affected the fertility of these heifers in the first service. Nutritional status during early pregnancy also appears to be critical, as moderate energy restriction in beef and dairy heifers from 10 days before conception until day 80 or 120 of gestation adversely affects ovarian development in their female offspring [17,38]. In addition, high maternal milk production during early gestation has been shown to be negatively associated with AMH concentrations in offspring at birth [39,40]. These findings suggest that prenatal conditions contribute to the marked inter-individual variability in ovarian reserve observed in cattle and support the concept of a physiological trade-off between maternal lactational demands and the establishment of the ovarian reserve.

When assessing ovarian reserve, an AMH blood test has an advantage over AFC determination in that it can be performed prior to the use of assisted reproductive techniques, regardless of the time, location, cycle, or personnel involved. For AMH measurement, a human ELISA kit was initially validated for cattle and successfully used in this species [8,41]. However, it was recognised early on that the development of species-specific tests would help to better differentiate between individuals within a species [42,43]. Therefore, different assay platforms have been employed, ranging from human AMH ELISAs with lower sensitivity and bovine under-reading [44,45], to automated platforms such as miniVIDAS [46] or Elecsys [47], to species-specific ELISAs [48,49]. With a specific bovine ELISA, approximately 44-fold higher AMH levels were measured than the mean and median AMH levels measured with the human ELISA kit, and a higher correlation between AMH and the average number of collected embryos was achieved [42]. Comparatively, human and equine specific AMH tests are being used in parallel in horses [28,29,30]. However, the different tests lead basically to the same conclusions in both species, albeit with varying degrees of accuracy.

In human reproductive medicine, AMH has developed into a parameter with a broad spectrum in clinical diagnostics, mainly based on its suitability for assessing the number of antral and preantral follicles in a patient. In women, fertility and suitability for assisted reproduction is often estimated with a triplet of AMH, AFC, and age. Low serum AMH concentrations indicate a limited ovarian functional reserve and an associated low response to hormone treatment. AMH levels are also used in women to determine the individual FSH dose for ovarian stimulation therapy. This ensures optimal oocyte retrieval and also prevents hyperstimulation syndrome [11]. Moreover, AMH is used to diagnose (premature) menopause, granulosa cell tumours or polycystic ovary syndrome (PCOS) in women [11]. In veterinary medicine, AMH is primarily utilized for the diagnosis of granulosa cell tumours [50] or castration status [51,52] but has not yet been incorporated into routine fertility assessment protocols. Therefore, the role of AMH in the regulation of follicular development and its practical relevance as an indicator of fertility and embryo production in cows and mares will be examined below.

## 2. AMH in the Regulation of Follicle Development

In cows and mares, AMH is mainly secreted by granulosa cells of primary and secondary follicles, as well as those of early antral follicles [12,53]. AMH-positive staining is absent in the ovaries at the time of sexual differentiation and is only being detected in foetuses after the occurrence of secondary and antral follicles [54,55]. After birth, ovarian AMH production is determined by the pool of small antral growing follicles and this is well reflected in plasma, but not follicular AMH levels [12,27,29]. In cattle, AMH inhibits activation and growth of ovarian follicles in vitro [55], suggesting that the follicles of the ovarian functional reserve can partially regulate their participation in the recruitment phase and later follicular wave. As shown in humans and mice, AMH seems able to attenuate FSH-induced follicle growth and steroidogenesis, most likely via modulation of oestrogen synthesis and desensitisation of the FSH receptor [5,56,57,58].

When the intrafollicular endocrine profile of mares was examined during antral follicle growth (Table 1), significant negative correlations were found between AMH and 17ß-estradiol concentrations, but not with progesterone [59]. Although only low concentrations of AMH are found in the follicular fluid of tertiary follicles in domestic animals, the dynamic changes in AMH secretion in antral follicles suggest a regulatory involvement of AMH in antral follicle development as an inhibitory factor in tertiary follicles [5]. In line with this, AMH was significantly elevated in equine follicles that exhibited steroid patterns of atretic follicles [60]. In contrast, the highest concentrations of AMH in cattle have been detected in the follicular fluid of small but viable follicles [5].

AMH is particularly expressed in cumulus cells and its levels increase in response to the cotreatment with the oocyte-secreted factors growth differentiation factor 9 (GDF9) and bone morphogenetic factor 15 (BMP15) [61]. Similarly, AMH expression is stimulated by GDF9 and BMP15 in mice granulosa cells and this effect is antagonized by FSH [62], suggesting a close interplay between the oocyte and the surrounding granulosa cells. Furthermore, human oocytes possess AMH receptor 2 and successfully mature in vitro in the presence of AMH, whereas coincubation with AMH and FSH lowers maturation [63]. The addition of AMH to the in vitro maturation medium can completely replace the gonadotropins FSH and hCG, as evidenced by up to 100% maturation rates of human oocytes solely with recombinant AMH [63]. In horses, slightly expanded cumulus–oocyte complexes (COCs), primarily from early atretic follicles, are considered to be particularly developmentally competent [59,64], while in cattle COCs with several compact cell layers, primarily from small vital follicles, are considered to be particularly developmentally competent [65]. Consequently, high AMH levels in the follicular fluid of early atretic follicles in horses [60] and in small healthy follicles in cattle [5] are entirely consistent with the developmental competence of oocytes and could represent species-specific characteristics.

The species-related peculiarities are of interest from the point of view of their potential significance for controlled superovulation in mares. In mares, ovarian superstimulation can only be induced with specific equine or recombinant equine FSH [66,67]. However, due to the anatomical characteristics of the mare’s ovary (ovulation fossa in the centre), superovulation with more than five preovulatory follicles on one ovary is counterproductive [67], as the additional preovulatory follicles can no longer reach the ovulation fossa, and too many ovulations lead to blood clots in the fallopian tube. Therefore, inducing double or triple ovulations in the context of in vivo embryo retrieval in mares appears to make more sense. The knowledge and targeted elimination of inhibitory factors such as AMH could therefore be helpful for the control of multi-ovulation. In mice, AMH treatment inhibits follicular development and ovulation, whereas treating mice with AMH prior to superovulation improves oocyte yield [68]. Thus, AMH may indeed be a potential therapeutic option for limiting or increasing ovulation rates in the future.

## 3. AMH as a Biomarker of Fertility in Cows and Mares

Classical fertility traits in cattle are influenced by numerous intrinsic and extrinsic factors, limiting their usefulness for genetic selection and motivating the research for independent, objective biomarkers. Anti-Müllerian hormone, as a mainly independent indicator of ovarian reserve, has therefore attracted considerable interest. It exhibits relatively high heritability in cattle, making it a promising target for selective breeding. Accumulating evidence suggests that AMH can partially predict fertility outcomes; however, reported associations remain heterogeneous. While several studies have identified weak or absent correlations between AMH and conventional fertility parameters [15,34,69,70,71], others have reported positive relationships with selected reproductive traits [72,73,74,75].

**Figure 2:**
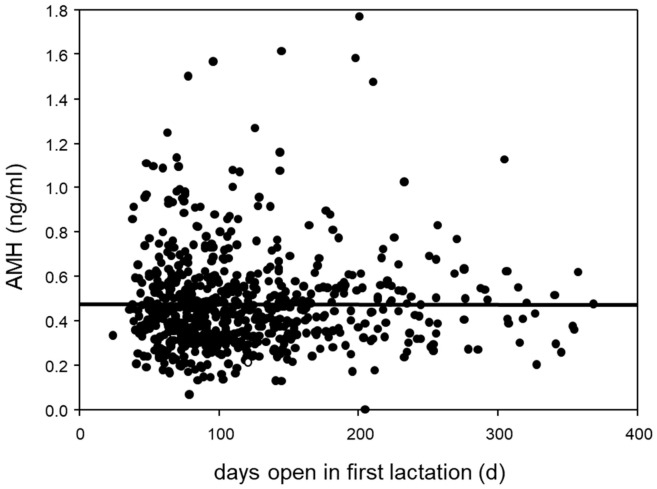
Days open of Holstein cows in their first lactation depending on their AMH concentrations as heifers before first service (scatter plot with linear regression). Simple analysis and short-term observations discovered no relationships between AMH and individual fertility in intensive milk production systems (according to Vernunft et al., 2018 [71]).

Part of this inconsistency appears to reflect context dependency. Large-scale evaluations in heifers have shown that AMH concentrations are largely independent of environmental and intrinsic biological influences, suggesting that AMH is a stable trait in heifers [36,71]. Nevertheless, a single AMH measurement in young animals has proven insufficient to reliably predict fertility under intensive production systems (Figure 2) [71]. In adult cows, associations between AMH and fertility are more apparent but depend strongly on management. Higher AMH is generally linked with improved overall fertility in systems relying on estrus detection or natural service, whereas its relationship with first-service conception under strict timed-AI protocols is weak or absent, likely because synchronization masks underlying biological differences [70]. Consistent with this, grazing Holsteins with intermediate or high AMH exhibit greater likelihood of pregnancy early in the breeding season [76], and Japanese Black cattle in the upper AMH quartiles show higher pregnancy rates and shorter intervals to conception [39].

Environmental and physiological challenges further modulate AMH–fertility relationships. Under heat stress, AMH remains associated with reproductive outcomes, but heat-induced impairment of estrus expression, ovulation, oocyte competence, and early embryonic survival can attenuate its predictive value [77]. In contrast, postpartum uterine and metabolic diseases do not appear to substantially affect AMH or AFC measured after calving, and higher AMH in pluriparous cows is still associated with shorter calving-to-first-service and calving to conception intervals, indicating resilience of AMH to short-term disease effects [75]. Interactions between AMH and milk production further complicate interpretation. In dairy cows, higher AMH is positively associated with milk yield, AFC, and estrus expression, particularly in older, high-producing animals [78], whereas other studies showed no relations to milk yield [71]. Therefore, negative correlations between milk yield and ovarian reserve are unlikely.

Evidence is strongest for long-term associations between AMH measured early in life and lifetime performance. Higher AMH in young heifers is positively associated with productive herd life, survival after first calving, cumulative pregnancy across lactations, and reduced culling for reproductive failure [69]. Conversely, heifers in the lowest AMH quartile exhibit shorter herd life, lower milk yield, reduced pregnancy rates, and increased reproductive culling. In beef heifers, higher prepubertal AMH is also associated with earlier puberty onset, reflecting accelerated activation of the hypothalamic–pituitary–gonadal axis and improved subsequent reproductive performance [16].

Beyond biological variation, analytical approach also influences detected associations. Linear models often fail to identify relationships between AMH and fertility, whereas quadratic analyses consistently indicate that animals with intermediate AMH values achieve the highest reproductive performance [72]. Group-based analyses support this non-linear pattern, with reduced fertility observed in animals at both low and high extremes of ovarian reserve [69,70,73,74,76].

The biological mechanisms underlying reduced fertility at the extremes of AMH appear to differ. Low ovarian reserve is consistently associated with impaired function across multiple components of the reproductive axis, encompassing the follicle, oocyte, early embryo, and uterus. At the ovarian level, low AFC is linked to compromised follicular microenvironments, characterized by altered nitric oxide signalling, reduced perifollicular vascularization, and increased oxidative and nitrosative stress, which together contribute to abnormal oocyte morphology and reduced developmental competence [79]. Concomitantly, granulosa cells from low AFC cows exhibit diminished responsiveness to gonadotropins, with reduced expression of FSH receptor, aromatase (CYP19A1), and AMH, resulting in lower estradiol production and attenuated steroidogenic capacity [80,81]. Beyond the ovary, diminished ovarian reserve is also associated with a less supportive uterine environment: beef heifers with low AFC display reduced concentrations of total and specific uterine luminal proteins, which are critical for early embryonic survival and development [82]. In contrast, recent evidence from high-AFC Angus heifers indicates that excessively rapid endometrial readiness, reflected by earlier luteolytic signaling, may be maladaptive, and that delayed endometrial PGF_2_α secretion is associated with improved fertility [83], highlighting that optimal reproductive success depends on coordinated ovarian–uterine timing rather than ovarian reserve alone. Collectively, these alterations provide mechanistic evidence that low ovarian reserve compromises fertility through reduced follicular sensitivity to gonadotropins, inferior oocyte quality, impaired endocrine support, and suboptimal uterine conditions for early pregnancy establishment. In contrast, reduced fertility in animals with very high ovarian reserve is less well understood but may involve altered steroidogenesis and elevated androgen production, resembling features of polycystic ovary syndrome in humans [84,85]. Consequently, although AMH is a robust indicator of ovarian reserve and lifetime reproductive potential, its causal role in determining fertility remains unresolved.

This topic has also attracted increasing interest in mares in recent years, but the results remain inconsistent. In a study of 419 thoroughbred mares, higher AMH concentrations were positively associated with fertility: pregnant mares had higher AMH levels than open mares, and pregnancy rates at days 13–18 post-mating increased with AMH concentration [25]. Moreover, mares in the lowest AMH quartile were significantly more likely to remain open, indicating reduced fertility compared with mares in the mid or upper AMH quartiles. In contrast, a study of 127 warmblood mares of varying ages and reproductive statuses found no associations between AFC or AMH and age-related fertility measures, reproductive status, number of cycles to achieve pregnancy, or seasonal pregnancy outcomes [30]. Similarly, in a cohort of 41 privately owned mares, early pregnancy rates assessed by ultrasonography 14–20 days post-ovulation were not associated with AMH concentrations, age, mare status, or semen type [13]. In this study, neither AMH nor AFC predicted the number of cycles required for conception or overall pregnancy outcomes. Taken together, these studies suggest that correlations between AMH and fertility in horses are generally weak, with potential predictive value limited to specific subgroups, such as older mares.

## 4. Significance of AMH for Embryo Production in Cows and Mares

It has long been described that AMH concentrations can be used as a predictive marker for embryo production in cattle via multiple ovulation embryo transfer (MOET) or oocyte pick-up (OPU) and in vitro embryo production OPU/IVEP. As shown early on in mice and humans, circulating AMH concentrations proportionally reflect the total number of oocytes and follicles in the ovary [7,9,11]. As the success of embryo production, via MOET or OPU/IVEP, is strongly dependent on the AFC at the start of reproductive programs [23], AMH has emerged as a logical prognostic marker for embryo yield.

Nearly two decades ago, it was demonstrated in cattle that AMH concentrations are indicative of an individual’s ability to respond to superovulatory treatments [23]. Since then, numerous studies in both dairy and beef cattle have addressed whether a reliable prediction of individual embryo collections can be made based on AMH levels.

The issue of sampling timing and frequency has also been addressed, with some authors analysing single or few samples taken shortly before superovulation (days to weeks) [32,44,45,46,47,86,87], while others performed repeated measurements over months or years to assess long-term stability and age effects [49,88]. In one case, a single juvenile measurement at 7–10 months of age was linked to embryo yield later in life [48], whereas in another study data collected over up to four years were included [44]. Regardless of breed, assay, age at sampling, or whether short- or long-term effects were analysed, most studies observed a positive association between AMH concentrations and the number of corpora lutea and embryos recovered after MOET [42,44,45,46,47,48,49,86,87,88,89,90,91].

Using quartile analyses to define low versus high responders, low AMH cows exhibited approximately half the number of corpora lutea and embryos compared to high AMH cows [86,87]. Moreover, reducing the FSH dose in high AMH donors further increased the number of transferable embryos, demonstrating that cows with a high ovarian reserve require, and benefit from, lower FSH doses [90]. This observation may partly explain why MOET efficiency has stagnated over recent decades and suggests that AMH could be used to adjust FSH regimens in the future. Results also demonstrate that within-animal AMH concentrations are moderately repeatable across cycles and over long periods, although they tend to decline with age and repeated superovulation [49].

Approximately half of the aforementioned studies also reported a correlation between AMH concentrations and the number of transferable or grade 1 embryos [44,47,86,87,88,90,91], which further enhances its potential applicability at the farm level. However, the prevailing consensus is that AMH predicts embryo quantity more than embryo quality.

In contrast, a limited number of studies, exclusively conducted in Holstein cows, reported no association between AMH concentrations and the response to superovulation treatment or subsequent embryo recovery [32,92,93]. These discrepancies may be attributable to small sample sizes or to homogeneity in AMH concentrations among enrolled animals, which can occur following long-term selection for fertility. While reliable prediction of individual embryo collections remains challenging due to the high biological variability, it is now evident that AMH can identify cows that are suitable donors on average. Furthermore, a threshold value of 74 pg/mL has been proposed for Holstein heifers, below which the use of a donor for MOET programs is not recommended [45]. Cut off values were also described for Gir heifers [94].

A consistent association has also been described between AMH concentrations and the quantity of aspirated oocytes and embryos produced in vitro, although its predictive strength as a cut-off tool varies across studies. This association was first described more than a decade ago, when Holstein (Bos taurus) and Nelore (Bos indicus) donors were evaluated and moderate correlations were observed between plasma AMH concentrations and the number of COCs recovered as well as in vitro produced embryos in both breeds [95]. Importantly, AMH did not influence blastocyst rate among recovered COCs, leading to the conclusion that AMH primarily reflects the size of the recruitable follicle pool and embryo yield rather than oocyte competence per se, and can be used to enrich for high embryo-producing donors.

Building on this foundation, we showed in Holstein heifers that AMH correlated positively with aspirated follicles, recovered oocytes, and embryos per OPU, though correlations, especially with embryo output, were modest [32]. Consequently, AMH was deemed insufficient as a precise predictor of per OPU performance but useful to classify very good versus very poor donors. Shortly thereafter, these observations extended to younger animals, demonstrating that Holstein and Nelore calves had higher AMH concentrations than cycling heifers and that AMH was strongly correlated with follicle numbers, COCs retrieved and cultured, and blastocyst production, independent of FSH superstimulation, highlighting AMH as a powerful tool for selecting elite calf donors with superior IVEP potential [96]. In parallel, in Hanwoo cows grouped by AMH level, higher COC numbers, blastocyst percentages, and embryos per cow were found in intermediate and high AMH groups compared with low AMH cows; embryos from intermediate AMH donors yielded the highest calf viability, although AMH did not directly predict pregnancy outcome per transfer [97].

Later studies reinforced the role of AMH as a stable, early-life selection marker across breeds and production systems. Japanese Black data demonstrated that a single AMH measurement in young heifers predicted future follicle and oocyte yield by OPU [98]. Similar relationships were confirmed in Wagyu cows, reporting higher AMH concentrations in high oocyte and embryo producers and proposing AMH measurement as a practical selection tool [99]. Most recently, Moon et al. showed in Hanwoo donors that AMH and AFC together improved prediction of oocyte yield and transferable embryos after stimulation, supporting their combined use in commercial programs [100]. Notably, AMH remained informative for donor selection even under dietary challenge, as low-level zearalenone contamination did not compromise AMH concentrations or oocyte quality [101].

Attention has more recently shifted from donor classification alone to optimizing hormonal management according to AMH phenotype, as the bovine IVEP market is continuously increasing. Building on preliminary observations from three decades ago suggesting a dose-dependent effect of FSH on OPU/IVEP outcomes in pregnant cows [102], a recent study has shown that administration of FSH prior to OPU enhances IVEP efficiency in both high- and low AMH pregnant heifers in a dose-dependent manner [103]. Notably, improvements in oocyte developmental competence reached a plateau at 280 IU of FSH in low-AMH but not high-AMH heifers, indicating that optimal FSH dosing to maximize IVEP outcomes may be lower for low-AMH phenotypes.

Given its value for donor stratification in cattle, AMH has increasingly been investigated as a potential indicator of oocyte and embryo yield in mares. Initial equine studies found a positive correlation between plasma AMH concentration and the number of aspirated follicles, but no correlation to the number of oocytes collected, most likely due to limitations of the aspiration technique used at that time [14]. More recent studies employing improved oocyte retrieval methods have clearly demonstrated that higher AMH levels in mares at the time of OPU are associated with a greater number of aspirated follicles, increased oocyte recovery, and a higher absolute number of embryos per OPU-ICSI session, especially when AMH concentrations exceed 2.5 µg/L [28]. However, AMH did not correlate with oocyte or embryo quality, including maturation, cleavage, or blastocyst rates. Considerable inter-mare variability was observed, and many mares with low-AMH concentrations still produced embryos, indicating that a single AMH measurement cannot serve as an independent predictor of OPU-ICSI success but can assist in identifying mares more likely to yield multiple embryos.

**Figure 3:**
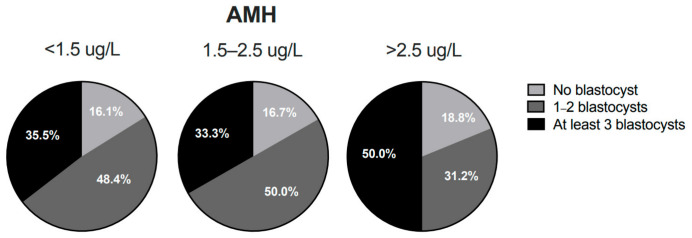
Outcome of an in vitro embryo production program grouped in no, one to two, and three or more embryos produced per session in mares assigned to low, intermediate, and high AMH concentrations (adapted from Scarlet et al., 2025 [104]).

Consistent with these findings, a very recent study showed that mares with higher AMH concentrations at the time of OPU were more likely to produce at least three embryos per OPU-ICSI session and suggested that AMH values above 3.3 µg/L were associated with an ~89% probability of yielding ≥3 embryos [104]. However, AMH concentrations did not differ between mares producing fewer than two embryos and those producing two or more embryos, further confirming that AMH is useful for identifying above-average embryo producers but not as a standalone predictor of overall success (Figure 3).

## 5. Conclusions

To date, AMH has not been incorporated into routine fertility assessment or treatment protocols in cows and mares. Nevertheless, growing evidence supports its functional role in follicle and oocyte development and highlights its potential utility in reproductive management and in vitro systems. While associations between AMH and fertility remain inconsistent—though optimal performance appears at intermediate levels and is more evident in cattle—AMH is a robust predictor of embryo production, primarily reflecting quantity rather than quality. Despite limited precision at the individual level, it can reliably identify high-performing donor groups (Table 2).

Taken together, AMH represents a promising biomarker for improving fertility assessment, optimizing embryo production, and guiding individualized FSH stimulation strategies. However, its application is currently constrained by biological variability and multiple influencing factors, including age, genetics, health status, and management conditions. Future research should therefore focus on standardizing analytical approaches and refining its integration into practical reproductive programs.

## Figures and Tables

**Table 1:** Concentrations of anti-Müllerian hormone (AMH), 17β-estradiol, and progesterone in equine follicular fluid depending on follicle size and function (According to Vernunft et al., 2012 [60] and Vernunft et al., 2013 [59]).

Follicle Diameter (Groups)	Concentrations in Follicular Fluid (LSMeans ± SE)			
(mm)	AMH (ng/mL)	17β-Estradiol (pg/mL)	Progesterone (ng/mL)	
8–17	164.3 ± 43.2 ^a^	131.1 ± 35.7 ^a^	29.8 ± 4.7	*n* = 15
18–27	32.5 ± 8.1 ^b^	238.4 ± 50.5 ^a^	29.4 ± 6.3	*n* = 16
≥28	4.9 ± 2.2 ^b^	771.1 ± 144.5 ^b^	24.4 ± 4.7 ^d^	*n* = 8
≥40 (preovulatory)	n.d.	1755.4 ± 187.8 ^c^	47.4 ± 16.5 ^e^	*n* = 5
8–17 atretic (E_2_/P_4_ < 2)	241 ± 79.6	39.9 ± 14.7	34.0 ± 9.2	*n* = 7
8–17 vital (E_2_/P_4_ > 2)	96.5 ± 29.5	210.8 ± 51.6	26.1 ± 3.7	*n* = 8

(n.d. = not detectable; Tukey–Kramer test: in columns: ^a,b,c^, *p* < 0.01; ^d,e^, *p* = 0.08). Follicular fluid was collected from the small (diameter 8–17 mm), medium (18–27 mm), and large (≥28 mm) follicle groups during diestrus. The preovulatory follicles were aspirated 28 h after ovulation induction with hCG during estrus. Follicles 8–17 mm in diameter were categorized according to their progesterone-estradiol ratio (E_2_/P_4_) in atretic (E_2_/P_4_ < 2) and vital (E_2_/P_4_ > 2).

**Table 2:** Utilisation of AMH as a parameter in different aspects of reproductive medicine in cows and mares.

Aspect	Cows	Mares
Main use in practice	Donor selection for MOET/OPU-IVP, heifer selection and culling decisions [44,47,69,86,87,88,91,96]	Identification of high-yield OPU-ICSI mares, assessment of ovarian reserve in subfertile or older mares [28]
Relationship to AFC/follicle numbers	Strong, generally linear across wide range; robust predictor of small antral follicle count [44,84]	Positive but more variable; strongest in mares with adequate baseline follicle numbers [14,26,29,30]
Embryo quantity in ART	High AMH → more follicles, oocytes, embryos per session [32,94,96,99,100]	High AMH → more oocytes and embryos per OPU-ICSI session [28,104]
Embryo quality/blastocyst rate	Reported associations are inconsistent [95,97,103]	Weak or absent association, with many low-AMH mares producing embryos [28,104]
Natural fertility outcomes	Moderate association with conception probability, herd life, pregnancy loss in some cohorts; context-dependent [15,34,69,70,71,72,73]	Limited data; AMH more established for ART yield than for natural pregnancy prediction [13,25,30]

## Data Availability

No new data were created or analysed in this study. Data sharing is not applicable to this article.
